# Food Waste in the Countries of the Gulf Cooperation Council: A Systematic Review

**DOI:** 10.3390/foods9040463

**Published:** 2020-04-08

**Authors:** Hamid El Bilali, Tarek Ben Hassen

**Affiliations:** 1 International Centre for Advanced Mediterranean Agronomic Studies (CIHEAM-Bari), Via Ceglie 9, 70010 Valenzano (Bari), Italy; elbilali@iamb.it; 2 Department of International Affairs, College of Arts and Sciences, Qatar University, Doha P.O. Box: 2713, Qatar

**Keywords:** bibliometrics, environment, food consumption patterns, food losses and waste, food waste, food wastage, food security, food waste recovery, Gulf Cooperation Council, waste management

## Abstract

Food waste (FW) is a critical challenge in the Gulf Cooperation Council (GCC). This paper analyzes research dealing with food waste in the GCC countries (viz. Bahrain, Kuwait, Oman, Qatar, Saudi Arabia, United Arab Emirates). It draws upon a systematic review performed on Scopus in January 2020. The paper covers both bibliometrics (e.g., authors, affiliations, journals) and research topics (e.g., causes, food supply chain stages, extent and quantity, food security, economic impacts, environmental implications, management strategies). A main finding of the review is the scarcity of data on FW in the GCC in general and in Kuwait, Oman, and Bahrain in particular. Most of the selected articles address FW reuse and recycling (e.g., waste-to-energy conversion, compost production). Indeed, other FW management strategies, such as reduction/prevention and redistribution, are overlooked. The systematic review highlights that further research on FW in the GCC is highly needed with a focus on the identified research gaps such as causes and drivers, trends, magnitude and extent, environmental and economic impacts, along with implications of food wastage in terms of food security. Since food wastage is a common issue for all GCC countries, these research gaps should be addressed in a shared regional research agenda.

## 1. Introduction

Food losses and waste (FLW) refers to “a decrease, at all stages of the food chain, from harvest to consumption in mass, of food that was originally intended for human consumption, regardless of the cause” [1]. FLW occurs between when an agri-food product is harvested and when it is consumed or discarded. Indeed, FLW occurs along the whole food supply chain from harvesting, transportation, storage, processing/packaging, distribution to consumption. Food waste may be considered as ‘food loss’ when occurring in the initial stages of the food supply chain (e.g., harvesting, transport, storage), and as ‘food waste’ when incurred within later stages e.g., retail and consumption [1,2,3]. There are significant differences among countries and from a commodity and season to another [1,2,3,4,5,6]. In particular, losses occurring in the initial part of the food supply chain (mainly caused by poor harvesting, transport and storage infrastructure, and facilities) are high in the developing world, whereas in developed countries, food waste mainly occurs within later stages i.e., in distribution and at consumer level [1,2,3].

FLW is an emerging issue with massive environmental, economic, and social implications [1,3,7,8,9]. Food wastage is also an ethical scandal [10] in times when more than 820 million people are still hungry worldwide [11]. Indeed, some scholars associate overconsumption and obesity to food waste [12]. Today, it is assessed that 1.2–2 billion tons (about a third of food produced worldwide for human consumption) is lost or wasted [1,2]. FLW undermines the foundation of food security [1,3,13,14,15]. When converted into calories, FLW amounts to about a quarter of all food produced at the global level [15,16,17]. FLW also represents a loss of valuable nutrients (including micro-nutrients) [18]. The reduction of FLW is also considered crucial to decrease the food-related environmental footprints [1,2,15,19,20,21]. Indeed, food waste amounts to a major depletion of resources (e.g., both natural resources, such land and water, and other economic resources, such as labor, energy, and capital) at global and local levels [2,15,22]. FLW represents, on the one hand, a waste of the resources utilized to produce wasted food and, on the other hand, a major source of negative environmental impacts including the emissions of greenhouse gas (GHG) that cause climate change [20]. Indeed, food waste is a significant contributor to global warming once in landfills [23]. Food waste represents the largest waste component sent to landfills and the primary source of landfill gas. Once food waste is landfilled, it decomposes under anaerobic conditions and generates methane emissions [24], a gas that is more than 25 times as potent as carbon dioxide (CO_2_) and that makes a significant contribution to global warming [20,25]. Besides carbon footprint (cf. GHG emissions) [26], food waste-related footprints include water footprint (cf. consumption of water resources) [27,28], ecological footprint (cf. use of agricultural land) [29,30], and nitrogen footprint [31]. Indirect externalities include the effects of intensive agriculture such as water pollution, deforestation, biodiversity loss, and land degradation (e.g., soil erosion, desertification) [32]. FLW implies the use of 1.4 billion ha of arable land, emission of 3.3 billion tons of CO_2_eq [20], and accounts for more than 25% of freshwater resources use as well as the yearly consumption of over 300 million barrels of oil worldwide [15,33,34]. Food waste also represents a considerable loss of money for all food supply chain actors, including producers and consumers [1,2,7,35,36]. Indeed, it translates into lost investment and income for producers and higher prices and food-related expenses for consumers [1,14,22]. In this respect, the Food and Agriculture Organization of the United Nations (FAO) [20] puts that FLW accounts for approximately 680 billion US$ in developed countries and 310 billion US$ in developing ones.

Many food waste management hierarchies or pyramids have been developed in the last years. These prioritize prevention and reduction at source and show a list of preferences for waste use, re-use, recycling, and treatment [1,37,38,39]. In general, all food use hierarchies prioritize FLW prevention and redistribution of food, that is still edible but cannot be sold before its expiry date, such as through food banks. In fact, food waste management hierarchies prioritize these two options (prevention and redistribution) with respect to using FLW as animal feed, for the production of compost and/or energy, or its disposal in landfill, which should be the last recourse [1]. 

Food waste is a serious issue also in the NENA (Near East and North Africa) region [40,41,42,43,44,45,46]. FAO [2] estimates that about 34% of food is lost or wasted across the region. Indeed, the amount of food waste is high in the NENA region [42,43,44,45,47] and aggravates food insecurity, scarcity of water, and environmental footprints/impacts while increasing food imports in a region that is already highly dependent on import [43,47,48,49]. Indeed, NENA countries suffer from a ‘double burden’ of malnutrition where problems of undernutrition coexist with those of over-nutrition (cf. obesity) [42]. Malnutrition and food insecurity are still important challenges in the NENA region [42], although there are important differences among poor, instable countries (e.g., Somalia, Yemen), and rich countries of the region such as those of the Gulf Cooperation Council (GCC) (viz. Saudi Arabia, United Arab Emirates (UAE), Kuwait, Oman, Qatar, Bahrain,) [50]. In particular, GCC (Gulf Cooperation Council) countries have a good food security status [51] but face numerous health challenges such as obesity [52], which is associated to unhealthy diets and lifestyles. Berjan et al. [41] show that FLW implies that from 265 m^3^/capita/year (Yemen) to 790 m^3^/capita/year (UAE) of water is wasted in NENA countries. In terms of food supply chain stages, more recent data [43] show that 32% of food waste in the NENA region occurs at the consumer level, while up to 68% occurs in the early stages of the food supply chain (viz. production, handling, processing, and distribution/retail). In high-income countries, such as those of the GCC, food is to a large extent wasted at the consumer level [1,2,4]. However, findings of different surveys highlight that household food wastage is also a serious issue in middle-income NENA countries such as Algeria [53], Egypt [54], Lebanon [55], Morocco [56], and Tunisia [57]. The most wasted food products in the NENA region are fruits/vegetables (45% of production), fish/seafood (28%), and roots/tubers (26%) [43,47]. 

Nevertheless, all figures reported above on FLW in the NENA region are only estimations as accurate data have not been systematically collected [47]. Indeed, Abiad and Meho [45] underline the “*paucity of applied studies that investigate the drivers, sources, management, quantification, policies, interventions, and initiatives to reduce food loss and food waste in the Arab world*” (p. 311). FAO Regional Office for the Near East [43] highlights a critical lack of information on FLW in the NENA region. However, quantitative data on the causes, magnitude, and food supply chain stages where food is wasted is vital to take remedial actions. Although research is needed to get reliable data on food waste, there has been so far no systematic study of the state of research on this issue in the NENA region in general and the countries of the GCC in particular. To bridge this knowledge gap, this review provides a comprehensive, systematic overview of the landscape of research on food waste in the GCC. It combines bibliometric and topical analyses to outline a profile of the research field, identify research gaps, and provide recommendations for strengthening the research strands on food waste in the countries of the GCC. 

## 2. Methods

**Figure 1:**
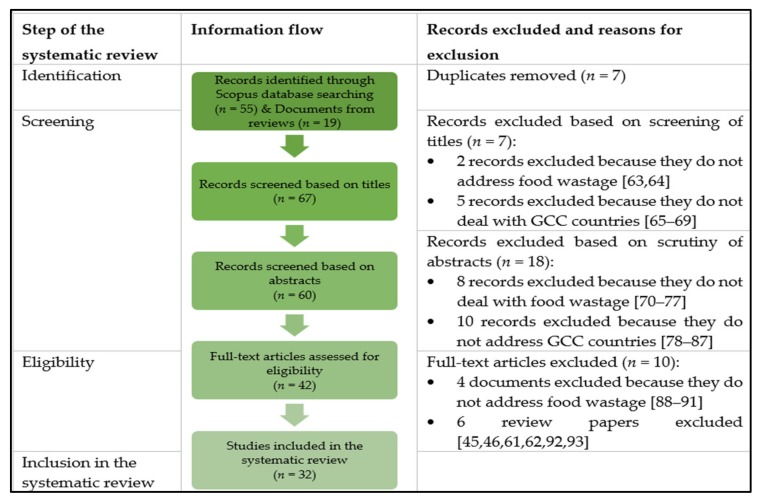
Process of the search and selection of documents, *n* = 7 [63,64,65,66,67,68,69], *n* = 18 [70,71,72,73,74,75,76,77,78,79,80,81,82,83,84,85,86,87], *n* = 10 [45,46,61,62,88,89,90,91,92,93].

The article draws upon a systematic review of records indexed in Scopus (Figure 1). The systematic review is in line with the PRISMA (Preferred Reporting Items for Systematic Reviews and Meta-Analyses) guidelines [58]. The methodology followed for documents selection is similar to that suggested by Moher et al. [58] and adopted by El Bilali [59,60]. A search was carried out on January 12th, 2020, using the *Title-Abs-Key* search query *(“food wastage” OR “food waste”) AND (Bahrain OR Kuwait OR Oman OR Qatar OR “Saudi Arabia” OR “United Arab Emirates” OR UAE OR “Gulf Cooperation Council” OR “Gulf countries” OR “Middle East” OR “Near East” OR “West Asia”)*. 

The initial search yielded 55 documents. A further 19 articles were added from some recent reviews on food waste in the NENA region [45,46,61,62]. The number of documents left after removing duplicates was 67. For the inclusion in the systematic review, a document had to meet simultaneously three criteria relating to thematic focus (viz. food waste is central topic in the document), geographical coverage (viz. the document deals with one or more GCC countries), and document type (viz. the document is a journal article, book chapter, or conference paper; grey literature such as reports and discussion papers, letters to editors and/or notes were excluded).

Following the screening of the titles, 7 records were excluded as they do not address food waste in the GCC countries. An additional 18 documents were not considered for further analysis based on the scrutiny of abstracts. At this step, also excluded were records that address only recycling or reuse of ‘unavoidable food waste’, that is to say inedible parts of agricultural and animal products which are, anyway, not suitable for human consumption such as camel bones [71]. A further 10 records were left out based on the analysis of full texts. Consequently, 32 documents were considered in the systematic review (Table 1) and underwent deeper analysis.

Both bibliographical metrics and topical focus of research on food waste in the GCC countries were addressed in the systematic analysis of available literature (Table 2). Qualitative analysis, synthesis and conceptualization of data from the selected documents were informed by Hsieh and Shannon [126] and Petticrew and Roberts [127].

This systematic review was not without limitations. In particular, as in any systematic review, the search process affects the results. First, the use of the Scopus database means that only quality scholarly, peer-reviewed literature was considered (e.g., research published in journals not indexed in Scopus and grey literature—such as reports—are not included in the present systematic review). Second, the selection of search terms also has an affect on the review results and this is the case also in this systematic review. In particular, while the terms ‘food waste’ and ‘food wastage’ often encompass also ‘losses’, the fact of not including specific terms such as ‘food loss’ or ‘postharvest loss’ in the search string might have implied that some documents which refer only to ‘losses’ might have been overlooked during the search and, consequently, not included in the present review. However, different synonyms were utilized to broaden the basis of the initial screening of records and broader geographical areas were considered (viz. Middle East, Near East, West Asia) in order not to miss out on any piece of research dealing with food waste in the countries of the GCC. 

## 3. Bibliographical Metrics of Research on Food Wastage in GCC Countries 

One of the main results of this systematic review is the marginality of research on FW in the GCC countries (viz. Saudi Arabia, UAE, Kuwait, Oman, Qatar, Bahrain). It is, in fact, surprising that only 32 documents resulted dealing with food wastage in the GCC given the magnitude and extent of the problem in the region and its tight link to food security. Indeed, the 31st regional conference of FAO in the Near East recommended to “*[…] assist member countries in addressing the key challenges of reducing food waste and losses by conducting comprehensive studies on the impact of food losses and waste on food security in the region and in establishing a plan to reduce food losses and waste in the region by 50% within 10 years*” (p. V) [43]. Most likely, it is for the awareness of the lack of data and studies on this issue that the Regional Strategic Framework on ‘Reducing Food Losses and Waste in the Near East and North Africa Region*’* devoted one out of its four components to ‘*Data gathering, analytical research, and knowledge generation*’ (p. 4,5) [43]. The need for further research on food wastage issue in the region was also highlighted by many scholars [40,41,45,46].

There are enormous differences among the GCC countries in terms of research dealing with food wastage. It seems that research on food waste is mainly performed in Saudi Arabia (Table 3). This is quite normal considering that the country is the largest and the most populous one in the GCC. In fact, it is important to take into consideration the sizes of countries and their research systems (e.g., country research performance is assessed on Elsevier’s SciVal using the number of scientific articles per million inhabitants as an indicator). However, also Qatar and, to a lesser extent, UAE are active in the research field on food waste. On the other hand, such research field is marginalized in Kuwait and Oman, while there was no paper dealing with food wastage in Bahrain. Surprisingly, although food wastage is a common issue for all GCC countries, there is no single study that addresses this issue in a comprehensive way in the whole region. 

Bibliographical metrics (journals/sources, subject areas, authors, affiliations/institutions, affiliation countries) of research addressing food waste in the countries of the GCC are presented in Table 4. 

Interest in food waste has been increasing steadily globally as well as in the GCC. However, despite the evident increase in research dealing with food waste in the countries of the GCC, research productivity and publication rate have been fluctuating from one year to the next. The output of articles per year ranged from nine (2018) to none in many years (e.g., 2010, 2011, 2008, 2005, 2002, 1999). The average output of articles in the period considered in the present systematic review (1987–2019) is less than one article per annum. According to Abiad and Meho [45], “*this deficiency in research productivity on FLW in the Arab world can be attributed to several possible factors that are not mutually exclusive, such as a lack of interest in the subject matter among local scientists; the small number of local scientists in the field of food loss and food waste; lack of funding and governmental support; and/or difficulties in carrying out such research in the Arab world as a result of cultural and religious barriers*” (p. 4).

As for journals and sources, results analysis shows that most of the selected articles were published in the *Journal of Cleaner Production* (3 papers), *Energy Procedia* (2 articles), and *Waste Management* (2 articles). Nevertheless, the findings of research on food wastage in the GCC countries were published in another 24 journals and sources. This clearly shows that, so far, there is no earmarked journal where most of the results of research on food waste in the GCC are published. Evidently, there is a relationship between journals and subject areas; so, it comes no surprise that most of the selected articles are categorized in the areas of environmental sciences (10 articles), engineering (9 articles), and science technology (7 articles). However, the selected articles can be classified in other 14 subject areas (e.g., computer science, agricultural sciences, educational science, behavioral sciences, food science and technology, information science, management science). This might explain how difficult it is to grasp the food wastage research field, which is rather multidisciplinary. Nevertheless, it can be put that the focus of research on food waste in the GCC countries is on engineering and environmental sciences, while social sciences and economics are largely overlooked. 

The authors that contributed the most to research on food waste in the GCC countries are Nizami A.S. (8 papers) and Rehan M. (5 papers). However, the analysis of the number of articles per author suggests that there is no consistency in this field of research. In other words, it might be that most of the researchers that deal with food wastage in the GCC do that in a sporadic, unsystematic way, which might indicate a lack of specialization in the field that, in turn, can be due to the absence of any medium- or long-term articulated, structured research project/program on food wastage in the region.

The analysis of author countries and affiliations suggests that Saudi authors and institutions perform better and are more productive in research on food wastage than the other GCC countries (viz. Bahrain, Kuwait, Oman, Qatar, UAE). Indeed, affiliation countries are dominated by Saudi Arabia (15 papers) followed by Qatar (8 papers) and UAE (5 papers). However, there are many international collaborations on this topic that involve researchers from Arab and Muslim countries (e.g., Pakistan, Egypt, Jordan, Malaysia), Europe (e.g., England, Austria, Belgium, Hungary, Poland, Sweden), Asia (e.g., India, Japan), North America (i.e., Canada, USA), and Oceania (e.g., Australia). Likewise, affiliations are dominated by Saudi research institutions and universities such as King Abdulaziz University (9 papers) and Prince Mohammad Bin Fahd University (3 papers). Contributors to the research field in the GCC countries also include Prince Mohammad bin Fahd University, Imam Abdulrahman Bin Faisal University, and King Faisal University in Saudi Arabia; Qatar University and Hamad Bin Khalifa University in Qatar; Khalifa University of Science Technology, United Arab Emirates University, and University of Sharjah in UAE; and Sultan Qaboos University in Oman. Meanwhile, it seems that no institution in Bahrain has published papers on food wastage. Nevertheless, it is worth mentioning that research on food waste in the GCC is also performed by other institutions outside the region (e.g., Cranfield University, UK; Brunel University, UK; Georgetown University, USA). 

## 4. Topics Addressed in Research on Food Waste in the Gulf Countries 

The following sub-sections describe and analyze the findings on food waste in the GCC countries from the reviewed literature with a particular focus on the causes of food wastage (4.1), stages of the food supply chain (4.2), extent and quantity of food waste (4.3), food wastage and food and nutrition security (4.4), food waste prevention and management strategies (4.5), economic impacts of food wastage (4.6), and environmental implications of food waste (4.7). Each sub-section includes an evaluation of the whole selected literature to see whether or not it addresses each specific topic (and to what extent) as well as an analysis of how the topic was addressed by comparing and discussing data from the different selected papers.

### 4.1. Causes of Food Wastage 

FAO [128] suggests that the causes of FLW vary across the NENA region and include inadequate and weak infrastructure (e.g., cold chain, markets), inappropriate regulatory and policy frameworks, and institutional weaknesses. Likewise, FAO [43] argues that poor farming systems, inappropriate postharvest practices (e.g., cold storage, handling, drying), and deficient infrastructure are among the main causes of food wastage in the region. While there is no paper that analyzes the causes of FLW along the whole food supply chain in the GCC countries, some papers address the drivers of food wastage in specific stages of the food change (mainly consumption) and/or in determined settings (e.g., university and hospital canteens, households). 

Baig et al. [46] argue that the “*key contributors to waste include culture, food valuation, policy and industry factors, and awareness*” (p. 1633) in Saudi Arabia. Likewise, Baig et al. [61] suggest that “*the factors responsible for food waste include: lack of awareness; insufficient and inappropriate planning when shopping. Food waste in restaurants, celebrations, social events and occasions is enormous. Waste is common in festivals and special events where the customs is to provide more food than required*” (p. 1743). In addition, Al-Zahrani and Baig [117] put that the reasons for food wastage comprise lack of awareness and lack of shopping planning. Focusing on food waste management (FWM) in Saudi Arabia (KSA), Mu’azu et al. [96] argue that “*major challenges to FWM in KSA include solid waste segregation, inadequate legislations, well accepted traditional landfill disposal practices, public attitudes, lack of awareness as well as uncertainty of FW byproducts acceptability*” (p. 678).

Abdelaal et al. [94] point out that, as far as the food outlets of a Qatari university campus are concerned, “*the root cause for the excessive food waste generation was overproduction rather than consumer wastage*” (p. 14), that is to say that too much food was offered on buffet, and highlight the need to focus in food waste reduction strategies also on raising the awareness of food providers, besides that of consumers. Al-Othman and Hewedy [123] found that the amount of food waste among residence students in Riyadh (Saudi Arabia) depended on the meal; it was higher for dinner (39.74% of the meal) than for lunch (22.67%). In their analysis of food wastage in the hospitality industry (e.g., hotels, restaurants) in Abu Dhabi (UAE), Pirani and Arafat [113] found that “*the factors contributing most significantly to food waste generation include serving style and timing, type of food served, and the prediction accuracy of the number of expected customers*” (p. 129). 

Following their investigation on the food waste behavior of consumers in Qatar, Aktas et al. [98] confirm that there are “*significant relationships between food waste and contextual factors such as motives, financial attitudes, planning routines, food surplus, social relationships and Ramadan*” (p. 658). Subsidies also contribute to wastage in general (e.g., water, energy) and food wastage in particular in the GCC countries [117,129]. In this respect, Irani et al. [101] highlight the strong linkages between food wastage and food-related policies that affect the management and efficiency of food supply chains. 

Anyway, many authors [45,96,101] pointed to the lack of accurate statistics and data about food wastage in the countries of the GCC and research should help in providing data on the magnitude and extent of food waste in the region. 

### 4.2. Stages of the Food Supply Chain 

Most of the selected articles address food wastage at the consumption stage, while no or little attention was devoted to remaining stages of the food supply chain, such as production and harvesting, storage, transport, and/or processing. This might be because most of food wastage in the rich GCC countries takes place at the consumer level. Indeed, Baig et al. [61] suggest that food is mainly wasted at the consumer level in Saudi Arabia; “*food is wasted at restaurants, caterers, cafeterias and, especially, by households such that food waste is the single-largest component of the landfills*” (p. 1743). Moreover, even at the consumption level, studies on behavioral and attitudinal aspects relating to food wastage are lacking; the only exception is Aktas et al. [98] who use TPB (theory of planned behavior) to investigate food waste behavior of Qatari consumers. The selected papers deal with food waste at consumption in different settings such as food outlets of university campuses [94,123], hospitals [109,124], hotels [113,114], as well as households [99,100,117,120,122].

Some studies highlight that food wastage increases in social and religious occasions such as the holy month of Ramadan and pilgrimage (Hajj) [45,98,110,111,117,118]. The increase in food wastage during the fasting month of Ramadan is ascribed to the preparation of meals that largely exceed families’ needs, although this is not conforming with the Islamic teachings, which prohibits wastage in every life aspect [45]. These findings regarding the relation between Ramadan and food wastage behavior are corroborated by the results obtained in other NENA countries e.g., Algeria [53], Egypt [54], Morocco [56], and Tunisia [57]. Food wastage is also high during social events in the Gulf countries [61,113,117]; Abiad and Meho [45] put that “*during social events, such as weddings, births, and deaths, food is usually prepared on a large scale, in many cases turning into lavish shows flaunting wealth and social status*” (p. 7). 

### 4.3. Extent and Quantity of Food Waste 

The selected articles do not encompass any comprehensive analysis of the quantity and magnitude of food wastage in the GCC countries. Moreover, the few presented figures do not make any distinction between edible and inedible (cf. unavoidable) parts of food. However, many studies show that the countries of the GCC are amidst the top world food wasters. Baig et al. [46] point out that the KSA might have one of the highest rates of food waste in the world and put that “*estimates of annual per capita waste of food ranged from 165 kg to 511 kg*” (p. 1633). Meanwhile, referring to data from the Food Sustainability Index [130], Baig et al. [61] argue that “*with 427 kg of food wasted per capita per year, the country ranks among the top food wasters*” (p. 1743). Shahzad et al. [110] highlight that FW is the largest stream in the municipal solid waste (MSW) in Makkah (Saudi Arabia) and represents slightly more than a half of MSW in the city. Bennbaia et al. [99] suggest that “*Qatar is one of the top 10 countries in the world in terms of per capita food waste; which ranges from 584 to 657 kg per year*” (p. 2495). 

In their investigation of food waste in the food outlets of a Qatari university campus, Abdelaal et al. [94] highlight that “*Food waste generation at the sampled locations was estimated at 329.5 kg/day or 80 t/year. Based on per sales estimates, total food waste was 980 g/sale and 757 g/sale at the student male and female housing complexes, respectively, equating to roughly one wasted meal for each sold meal*” (p. 14) and argue that overproduction, rather than student wastage, was the main driver of food wastage. They also underline that food providers lack the proper tools to assess food waste that is generated at their outlets/cafeterias. Regarding food wastage in hospitals, Al-Shoshan [124] report that food wastage at a Saudi hospital amounts on average to 24%−32% of the presented portions, which corresponds to 0.659−0.852 kg per patient and per day (cf. three meals: breakfast, lunch, and dinner). Concerning households, Aljamal and Bagneid [120] highlight that household food waste is high in Kuwait as about 60% of Kuwaiti households report they regularly waste food. Abu Qdais et al. [122] found that food waste represents approximately 50% of municipal solid waste (viz. 1.76 kg per capita per day) in residential areas of Abu Dhabi (UAE), which means that, already back in 1997, the average food waste of each Emirati was about 321 kg per year. 

### 4.4. Food Wastage and Food and Nutrition Security

The relationship between food waste and food security is particularly relevant in the NENA region [41]. Indeed, the region has a huge food deficit and depends heavily on food imports to satisfy the total food requirements of its population [43,47,48,49]. Hence, it is intolerable that the NENA region wastes each year up to 250 kg per capita, which is even more than the global food waste average [43]. Despite that, the relation between food wastage and food security is not explicitly addressed in the selected documents. However, many authors refer to food security to justify the need to reduce food wastage. 

Referring to the Kingdom of Saudi Arabia (KSA), Baig et al. [46] put that “*the KSA has limited arable lands and scarce water and thus relies on extensive imports and food subsidies to meet food demand. Accordingly, waste and loss of food are a significant concern for food security*” (p. 1633). This argument is valid for all the other five countries of the GCC. In addition, Baig et al. [61] highlight that food waste is one of the main issues that threaten long-term food security in Saudi Arabia. 

Al-Ali Mustafa [108] highlights the high reliance of Qatar on imports to feed its rapidly increasing population and underlines that no genuine sustainable food security can be attained without addressing rampant food waste in the country. Indeed, the reduction of food wastage has been side-lined so far in strategies to attain sustainable food security in Qatar. The increasing reliance of Qatar on food imports is driven not only by population growth but also by the development of the tourism sector, which has significantly increased not only the amount of food that is consumed but also that of food wasted in the GCC [114]. Therefore, Pirani and Arafat [114] suggest that “*the way forward for the GCC countries must involve diversified food supplies along with decreasing food demand in the first place*” also through the reduction of food wastage in all sectors (tourism sector included). One way to reduce food demand is to promote the shift of the population towards sustainable diets. In this respect, it is of paramount importance to develop national dietary guidelines that incorporate food sustainability principles (including food waste reduction) such as in the case of Qatar [116]. Indeed, unsustainable food consumption patterns and diets are drivers of high obesity rates, and per capita water use rates and waste footprints [88].

Irani et al. [101] show that the reduction of food losses and waste is essential to ensure long-term food security in Qatar. They highlight that factors contributing to food waste reduction are enablers to promote food security. In this context, Irani et al. [101] suggest that “*interventions to manage and mitigate the effects of food security and food waste should include knowledge transfer, market access and wider organisational involvement interventions”* (p. 381).

### 4.5. Food Waste Prevention and Management Strategies 

The considered papers enumerate different food waste recovery strategies ranging from food waste prevention to food surplus redistribution and food waste reuse (e.g., compost, energy) (Table 5). 

Papers dealing with prevention and reduction focus on how to address the root causes of food wastage as well as avoidance strategies. Irani et al. [101] analyze how big data can help in the management of food security in Qatar through the reduction of food losses and waste. In particular, they investigate a set of organizational factors to understand where and how food wastage occurs across the food supply chain. The authors show that various organizational factors (e.g., food market competition; standardized food regulations; bureaucracy level; working with local charities; creating one unified food authority; collaboration among food authorities and with food supply chain actors) may help in reducing food waste. Irani et al. [101] also argue that “*education and training of stakeholders in the food chain […] may inherently contribute towards the impact on the reduction of food waste. In doing so, promoting an increase in the recycling of food waste […] and thus better food quality management*” (p. 381). Al-Zahrani and Baig [117] highlight the importance of and need for public awareness on food waste in the KSA as well as the role that can be played by extension services in this respect. They argue that “*food waste can be reduced significantly by increasing public awareness on the food and water situation in the Kingdom through a national comprehensive campaign and vibrant extension education programs*”. Capone et al. [40] put that “*urgent actions are needed to raise the awareness of Arab consumers about this phenomenon. Cultural background should be exploited in awareness campaigns. Moreover, governments should speed up food support policy reform*” (p. 40). Indeed, to reduce food wastage while promoting food supply chain efficiency and fair food access, policy makers should eliminate perverse subsidies that foster unsustainable food consumption patterns [40,131,132].

As for the hospitality sector, Pirani and Arafat [113] recommend different food waste minimization strategies such as converting buffets to a la carte service, improving communication and cooperation between the staff of hotel/restaurant and the guests, and encouraging hotels and restaurants to donate surplus food from buffets of events to local charities and food banks.

Traditionally, strategies to achieve food security in NENA countries have been centered on boosting agriculture and food production, whereas much less stress has been put on measures for the reduction of FLW. Measures addressing FLW, if implemented properly, can help enhance food security status in the region while reducing pressure on its scarce land and water resources [128]. Nevertheless, addressing food wastage requires considering the whole food supply chain, hence a deep understanding of food systems [133,134] is needed. 

As the causes of food wastage are different, also solutions depend on the stage of the food supply chain and differ among world countries and regions. These solutions should be implemented at different macro, meso, and micro levels; from global to national, local, and household levels [1] and along the whole food supply chain. In this respect, the integration of food waste reduction into policy is crucial. Such integration can take place in two different ways; either integrating FW concerns in all relevant sectoral policies or devising a specific, ad-hoc FW reduction policy. Furthermore, the efficiency of FW reduction solutions depends on the participation of a wide array of food supply chain stakeholders from public, private, and civil society sectors [1]. Therefore, FW reduction also implies new ways of organization and governance of food supply chains [135]. Indeed, reducing FW is a multi-factorial, multi-sectorial, multi-disciplinary, and multi-stakeholder endeavor, which entails coordination and networking among various actors and stakeholders. Regulations and policies are key to reduce FW alongside the effective participation of all relevant actors [43]. Consumers play a vital role in the reduction of food wastage in rich countries such as those of the GCC. According to Quested et al. [136] measures that consumers can put in practice for the reduction of food waste comprise: using shopping lists to better plan purchases, thus avoiding buying more than is needed as well as impulsive purchase of food that is not required; making more precise evaluation of portions to be prepared; understanding distinction between label dates (viz. ‘use by’ vs. ‘best before’); improving stock management and storage practices; enhancing knowledge on the use of leftovers.

Numerous authors deal simultaneously with different food waste management strategies. For example, Mu’azu et al. [96] deal with food waste redistribution and recycling. As for food waste redistribution, they put that “*recent Saudi Food Bank initiative has been recording tremendous successes in FW source reduction, though more stakeholders’ efforts are required while government proactive support and policies are necessary to ensure prosperity of such types of initiatives*” (p. 678). Likewise, Irani et al. [101] address all food waste management strategies in the context of achieving long-term food security in Qatar. Concerning food waste recycling, which is strongly linked to the concept of circular economy, Mu’azu et al. [96] argue that “*taking into cognizance the global trend of concept of circular economy with immense contributions of FW to prospective bio-refineries could be of paramount importance in achieving environmental sustainability as enshrined in KSA Vision-2030*” (p. 678). 

Food waste represents a promising source of raw materials to produce different fuels and chemicals. Various authors analyze the recycling of food waste into products such as biofuels, biochar, or compost [90,91,95,97,100,102,104,105,106,107,110,111,112,115,119,125,137]. For example, Elkhalifa et al. [95] investigate the pyrolysis of food waste for the production of biochar in the Qatari context and add that “*the produced chars can be utilized in carbon sequestration when applied as soil amendment and as precursors for higher value-added products such as adsorbents*” (p. 901). Technologies for the recovery of energy include incineration, anaerobic digestion (AD), transesterification, pyrolysis, and RDF (refuse derived fuel) [111]. Abdallah et al. [97] show the financial feasibility of waste-to-energy integrated strategies (viz. anaerobic digestion, incineration) in the UAE. Rehan et al. [102] assess the production of biodiesel from waste sources that are available in the KSA and highlight that conversion of food waste into biodiesel can not only contribute to energy generation but also solve many problems relating to waste disposal. They highlight that biodiesel production from waste sources in Saudi Arabia could produce around 1.08−1.41 mln tons of biodiesel with an energy potential of 43423−56493 TJ by 2030. In addition, Baawain et al. [107], after analyzing the composition of the MSW in Muscat (Oman), recommend a ‘waste-to-energy’ program given its high energy content. As converting food waste into compost, Waqas et al. [103] highlight that “*in KSA food waste is the most abundant stream of municipal solid waste that contribute up to 50% of the total waste*” (p. 426) and analyze the optimization of the process of compost production from food waste in Saudi Arabia. Likewise, Natour [125] suggests that over 75% of municipal and household waste in Kuwait city is compostable and that organic waste contains over 50% of food waste. Organic, compostable waste includes food waste from different sources like restaurants, households, hotels, canteens [87]. Al-Maaded et al. [91] put that 57% of MSW in Qatar is organic, hence compostable.

### 4.6. Economic Impacts of Food Wastage

The economic impacts of food wastage depend on the amount of money spent on food. As for 2018, the yearly expenditure on food (including non-alcoholic beverages) per capita in the GCC countries ranged from 1951.9 US$ (13.71% of total expenditure) in UAE to 1910.5 US$ (19.22%) in Kuwait, 1755.7 US$ (12.27%) in Qatar, 1689.6 US$ (20.62%) in Saudi Arabia, 1674.1 US$ (13.24%) in Bahrain, and 1329.1 US$ (22.65%) in Oman [138].

The analysis of the literature included in the systematic review shows that no document examines the impacts of food wastage on the prices of agri-food products in the GCC countries and how such changes in prices affect producers and consumers. Nevertheless, some papers estimate the financial value of food wasted in different settings (e.g., hospitals) as well as the economic benefits of recycling food waste (cf. energy, compost).

Al-Shoshan [124] estimates the monetary value of food waste over 2 days (i.e., six meals served to 759 persons among patients and attendants) in 18 general hospitals in Saudi Arabia and puts that the “*average plate waste represented approximately 40% of the meal cost/participant/day, and the estimated annual monetary loss for the 5.625 million regular meals to be served to patients and attendants will be around 35 million Saudi Riyals*” (p. 7), so about 9.3 million USD (as of February 1992). 

Other authors analyzed the economic benefits of the recycling and re-use of food waste. Abdallah et al. [97] compare the financial feasibility of anaerobic digestion and incineration, as waste-to-energy strategies, in UAE and highlight that while both strategies are financially feasible; “*the incineration strategy was more financially favorable in terms of the payback period, internal rate of return and profitability index, mainly due to the larger amount of processed waste*” (p. 207). Ouda et al. [112] compare two waste-to-energy (WTE) technologies, viz. incineration and RDF through ‘biomethanation’ (i.e., process of converting microbiologically organic material into methane under anaerobic conditions), in Saudi Arabia (KSA) and put that “*Biomethanation technology […] proved to be the most suitable WTE technology for KSA due to (a) availability of high food waste volume (37% of total MSW) that can be used as a feedstock, (b) higher efficiency (25-221230%) and (c) lowest annual capital ($0.1-0.14/ton) and operational cost*” (p. 328), but the need for a large space for operation and high labor skills requirements might limit the use of biomethanation. Nizami et al. [115], referring to MSW in KSA, put that “*the waste is highly organic (up to 72%) in nature and food waste covers 50.6% of it*” (p. 337) thus calculating that “*an estimated electricity potential of 2.99 TWh can be generated annually, if all of the food waste is utilized in anaerobic digestion (AD) facilities*” (p. 337). Shahzad et al. [110], focusing on biodiesel production from municipal waste in Makkah (Saudi Arabia), estimate that “*the cumulative net savings from landfill waste diversion (256 to 533 million Saudi Riyal (SAR)), carbon credits (46 to 96 million SAR), fuel savings (146 to 303 million SAR) and electricity generation (273 to 569 million SAR) have a potential to add a total net revenue of 611 to 1274 million SAR every year to the Saudi economy, from 2014 to 2050 respectively*”. Meanwhile, Rehan et al. [111] put that “*in Saudi Arabia and particularly in Holiest cities of Makkah and Madinah the benefits of waste to energy are several, including the development of renewable-energy, solving MSW problems, new businesses, and job creation and improving environmental and public health*” (p. 688).

Resource recovery through composting can bring about significant environmental and economic benefits. Waqas et al. [103] suggest that “*diverting food waste from landfills to optimized composting facilities using natural zeolites and biochar could benefit the KSA economy with a total net savings of about US $70.72 million per year*” (p. 426). Likewise, in their study on continuous thermophilic composting (CTC), Waqas et al. [104] argue that “*CTC can be implemented as a novel method for rapid decomposition of food waste into a stable organic fertilizer in the given hot climatic conditions of KSA and other Gulf countries with a total net saving of around US $70.72 million per year*” (p. 5212). 

### 4.7. Environmental Implications of Food Waste

There is no paper that analyzes in a comprehensive way the footprints of food wastage (e.g., ecological footprint, water footprint, carbon footprint, nitrogen footprint) in the GCC countries. Moreover, no paper deals with neither the relationship between food waste and climate change nor the indirect impacts of wasting food such as pollution of water resources as well as biodiversity loss, deforestation, and ecosystem disturbance. Nevertheless, Alruqai [121] puts that “*increased food waste production in major cities of Saudi Arabia is a challenge to environmental pollution*” (p. 230) and carries out a study on gaseous pollutants from food waste samples (containing mostly bakery products, rice, and meat) collected from restaurants in a public establishment in Riyadh (Saudi Arabia). He found that the production of methane was significantly higher than the production of other gaseous pollutants (e.g., ammonia, hydrogen sulfide). Based on that, the author recommends “*safe disposal of food waste in Riyadh, Saudi Arabia, in order to mitigate the environmental pollution resulting from land disposal of food waste in and around the capital*” (p. 230), as well as recycling of food waste in the KSA. 

The recycling of food waste has different direct and indirect implications [95,96,121]. Direct implications include the reduction of the quantity of waste that ends up in landfill [89,90]. Indirect benefits regard the use of some products from food waste such as biochar [95] and compost [90,91,103,105,106,119,125,137] which can not only improve soil fertility but also increase the carbon sequestration potential of soils, thus contributing to the mitigation of climate change. Indeed, soils in the GCC countries are mostly infertile [139,140] and have low organic matter contents (generally less than 5 g kg^−1^), which is, inter alia, due to the prevailing arid conditions combined with high temperatures that speed up soil organic carbon (SOC) decomposition and mineralization [140]. Food waste can also be used to produce environmentally friendly sources of energy such as biodiesels and biofuels [97,102,110,111,112,115]. Shahzad et al. [110] argue that the disposal of food waste in the landfills without any treatment represents not only a missed opportunity for energy recovery but also results in “*greenhouse gas (GHG) emissions and contamination of the soil and water bodies along with leachate and odors […] occurring in waste disposal vicinities*”. Bennbaia et al. [100] put that “*the use of recycled food waste as compost improves the soil health and structure, increases drought resistance and reduces the need for supplemental water, fertilizers and pesticides*” (p. 1340). 

Regarding water use, Seguela et al. [109] put that the processing of food waste in a health facility in Abu Dhabi (UAE) represents an opportunity to conserve water, thus reducing environmental impact through increasing waste recycling rate and decreasing desalinated potable water consumption. These benefits are vital in the desert, arid climate of the UAE. Indeed, effluent produced from food waste by dehydrators can be used for landscape irrigation. Pirani and Arafat [113] calculate the carbon and water footprints of food wasted at some events in Abu Dhabi (UAE). They point out that the average footprint of food waste of a lunch buffet is 4775 m^3^/guest for water footprint and 9.38 g CO_2_e/guest for carbon footprint. Meanwhile, the water footprint of a wedding buffet (for 300 guests) is 0.6 million liters of water, which is sufficient to meet the daily needs of about 17,000 persons.

## 5. Conclusions

Cutting the quantity of food waste is a concrete starting point to move towards sustainable food consumption and production in the GCC countries. Due to limitations of domestic agricultural production—in terms of arable land, water, and climate—the only way to close the widening production–consumption gap and meet the growing food demand of its affluent population has been the increasing dependence of the region on external food supplies. However, although the six GCC countries depend worryingly on food imports to meet their growing food demand, they stand out, paradoxically, amongst the world’s top food wasters. In fact, they waste about a third of imported food and their per capita food waste is even higher than in developed countries in North America and Europe. This might be, inter alia, due to the hot climate (that speeds up the deterioration of the quality of products) as well as the fact that most products are imported so with shorter shelf-life when they reach GCC countries because of the time needed for transportation, especially for fresh produce in case of dysfunctions in the cold chain. The high levels of food wastage across the GCC are alarming as they rise dependence on food imports and increase pressure on the scarce natural resources of the region. In particular, the wastage of domestically produced food implies the waste of natural resources such as water and arable land (including scarce soil nutrients), that are even much more precious in the GCC region than elsewhere and determines an inefficient use of available domestic resources. Therefore, FLW reduction is crucial to achieve long-term food security and food sustainability in the region. Furthermore, the reduction of FLW is vital to decrease pressure on the scarce natural resources (especially water). 

This is, to the best of our knowledge, the first systematic review on food waste in the GCC. Its relevance lies not only in informing food supply chain actors (including policy makers, scientists, etc.) on the landscape of research on food waste in the GCC, but also in synthesizing available data and research findings, pointing out the existing knowledge gaps, and identifying needs in the research field. The systematic review of literature clearly shows that research on food waste/wastage is still marginal in the GCC in general and in Kuwait, Oman, and Bahrain in particular. Moreover, the analysis of the available specialized literature shows that research focuses on food waste recycling and re-use while reduction, prevention, and redistribution strategies are often overlooked. Research gaps also include causes and drivers of food wastage, extent and magnitude of food waste along the whole food supply chain, implications of food wastage for food and nutrition security, economic impacts of food wastage, and environmental footprints of food waste (e.g., carbon footprint, water footprint, ecological footprint, water and air pollution, climate change). Future research should also pay more attention to the type of wasted food as it determines not only environmental and economic footprints of food wastage but also food waste management strategies (viz. prevention, redistribution, reuse, and recycling). While food waste prevention and avoidance should be given priority, as well as research on food waste behaviors among Gulf consumers, it is also vital to put more emphasis on strategies relating to the use of food waste as animal feed, including analysis of linkages with food safety legislation. Given the magnitude of food wasted in the GCC countries and the obvious evidence and knowledge gaps, there is a huge need for further research to inform future policy and action for the reduction of food wastage in the region. The systematic review highlights that additional research on food waste in the GCC is highly needed with an emphasis on causes and drivers (attitudinal/behavioral, social/cultural, political/institutional, technological, etc.), trends, magnitude and extent as well as economic and ecological implications alongside policy strategies. The generation of new data through research is indispensable for designing effective and efficient policy actions and coping strategies towards addressing food waste issue in the Gulf countries. Therefore, more attention should be devoted to food wastage in the research policies and strategies of the GCC countries. Since food waste is a common challenge for all the GCC countries, addressing this issue entails a comprehensive, shared, and integrated regional research agenda that is appropriately supported by policy interventions. 

Research, policy, and action should be well coordinated to achieve sustainable outcomes. There is also a need for a better collaboration between research teams in the region. Research on food waste can be carried out through collaboration between research centers and education/academic institutions across the GCC. In this respect, an idea would be to implement in the GCC a similar joint research on the FLW topic as that coordinated by the Nordic Council of Ministers (viz. Sweden, Denmark, Norway, and Finland) [141]. Transdisciplinary research, with the active involvement of relevant food supply chain actors, is highly recommended in the future. Besides further research, also knowledge sharing is greatly needed. Indeed, research efforts should be coupled with the development and/or strengthening of knowledge-sharing platforms to facilitate the dissemination of research findings to the scientific community, policy makers, and the general public in order to provide evidence for the design of policies and guidelines and a setting for awareness raising campaigns, training, and capacity building activities that aim at reducing and/or preventing food wastage in the GCC countries. Such activities should capitalize and further strengthen recent, promising developments in this area and the increasing awareness about this issue in the region. For instance, Saudi Arabia performed a FLW Baseline in the framework of its National Program for Reducing Food Loss and Waste [142] and included ‘improving global water management, and reducing global food loss and waste’ in the agenda of its G20 presidency term [143]. In this context, it is vital to alert Gulf consumers on the consequences of their unsustainable food consumption patterns and to reform food-related subsidies to deter wasteful consumption in all its forms, which determine not only food wastage (with all its environmental, economic, ethical, and social impacts) but also other problems such as obesity and non-communicable diseases.

## Figures and Tables

**Table 1:** Documents considered in the systematic review of literature on food waste in the Gulf Cooperation Council (GCC) countries.

Year	Number of Documents	References
2019	3	Abdelaal et al. [94]; Elkhalifa et al. [95]; Mu’azu et al. [96]
2018	9	Abdallah et al. [97]; Aktas et al. [98]; Bennbaia et al. [99]; Bennbaia et al. [100]; Irani et al. [101]; Rehan et al. [102]; Waqas et al. [103]; Waqas et al. [104]; Waqas et al. [105]
2017	6	Alhajhoj [106]; Baawain et al. [107]; Al-Ali Mustafa [108]; Seguela et al. [109]; Shahzad et al. [110]; Rehan et al. [111]
2016	3	Ouda et al. [112]; Pirani and Arafat [113]; Pirani and Arafat [114]
2015	2	Nizami et al. [115]; Seed [116]
2014	1	Al-Zahrani and Baig [117]
2013	2	Amara et al. [118]; Khan and Kaneesamkandi [119]
2012	2	Aljamal and Bagnied [120]; Alruqai [121]
1997	2	Abu Qdais et al. [122]; Al-Othman and Hewedy [123]
1992	1	Al-Shoshan [124]
1987	1	Natour [125]

**Table 2:** Issues and topics analyzed in the systematic review.

Item	Elements Analysed
Bibliographical metrics and geography of research on food wastage in GCC	Metrics: sources/journals, authors, institutions/affiliations, subject areas Research geography: GCC countries considered or overlooked
Topical focus of research on FW in GCC countries	Causes of food wastage
	Stages of the food supply chain (viz. production, processing, distribution/trade/retail, consumption)
	Extent and magnitude of food waste
	Food wastage and food and nutrition security
	Economic impacts of food wastage
	Environmental implications of FW
	Food waste management strategies (e.g., prevention, redistribution, recycling and re-use)

**Table 3:** GCC countries where research on food waste was performed.

GCC Country (Number of Articles)	References
Kuwait (2)	Aljamal and Bagnied [120]; Natour [125]
Oman (1)	Baawain et al. [107]
Qatar (8)	Abdelaal et al. [94]; Aktas et al. [98]; Bennbaia et al. [100]; Bennbaia et al. [99]; Elkhalifa et al. [95]; Irani et al. [101]; Al-Ali Mustafa [108]; Seed [116]
Saudi Arabia (16)	Alhajhoj [106]; Al-Othman and Hewedy [123]; Alruqai [121]; Al-Shoshan [124]; Al-Zahrani and Baig [117]; Amara et al. [118]; Khan and Kaneesamkandi [119]; Mu’azu et al. [96]; Nizami et al. [115]; Ouda et al. [112]; Rehan et al. [102]; Rehan et al. [111]; Shahzad et al. [110]; Waqas et al. [103]; Waqas et al. [104]; Waqas et al. [105]
UAE (4)	Abdallah et al. [97]; Abu Qdais et al. [122]; Pirani and Arafat [113]; Seguela et al. [109]

**Table 4:** Metrics of research on food waste in the GCC: top ten subject areas, journals, authors, affiliation countries, and affiliation institutions.

Journals (a*)	Subject Areas (b*)	Authors (c*)	Affiliation Countries/Territories (d*)	Affiliation Institutions (e*)
Journal of Cleaner Production (3)	Environmental sciences (10)	Nizami A.S. (8)	Saudi Arabia (15)	King Abdulaziz University (9)
Energy Procedia (2)	Engineering (9)	Rehan M. (5)	Qatar (8)	Prince Mohammad Bin Fahd University (3)
Waste Management (2)	Science technology (7)	Ismail I.M.I. (4)	UAE (5)	Brunel University (2)
Bioresource Technology (1)	Energy science (4)	Ouda O.K.M. (4)	England (4)	Central Metallurgical Research Development Institute (CMRDI) (2)
Chemical Engineering Transactions (1)	Computer science (3)	Waqas M. (3)	Pakistan (4)	Cranfield University (2)
Computer Aided Chemical Engineering (1)	Agricultural sciences 3	Aburiazaiza A.S. (2)	Australia (3)	Georgetown Univ. (2)
Computers Operations Research (1)	Nutrition dietetics (2)	Aktas E. (2)	Canada (2)	Hamad Bin Khalifa University Qatar (2)
Environmental Science and Pollution Research (1)	Educational science (2)	Arafat H.A. (2)	Egypt (2)	Khalifa University of Science Technology (2)
Global Food Security (1)	Behavioral sciences (1)	Barakat M.A. (2)	Jordan (2)	Qatar University (2)
Journal of Agriculture and Environment for International Development (1)	Business economics (1)	Gardy J. (2)	Malaysia (2)	Universiti Putra Malaysia (2)

* Figures in brackets refer to the number of documents by (**a**) Journal, (**b**) Subject area, (**c**) Author, (**d**) Country/Territory, or (**e**) Institution.

**Table 5:** Food waste recovery and management strategies analyzed in the literature on food waste in the GCC.

Food Waste Recovery Strategy	References
Food waste prevention and/or reduction	Abdelaal et al. [94]; Aktas et al. [98]; Aljamal and Bagnied [120]; Al-Zahrani and Baig [117]; Amara et al. [118]; Al-Shoshan et al. [124]; Al-Othman and Hewedy [123]; Irani [101]; Pirani and Arafat [113]; Pirani and Arafat [114]; Seed [116]
Food redistribution (donation of extra/surplus food to food banks, soup kitchens, shelters, etc.)	Mu’azu et al. [96]; Irani [101]
Food waste recycling and reuse (e.g., animal feed, composting, industrial uses such as production of biofuel and oils)	Abdallah et al. [97]; Alhajhoj [106]; Alruqai [121]; Baawain et al. [107]; Bennbaia et al. [99]; Bennbaia et al. [100]; Elkhalifa et al. [95]; Irani [101]; Khan and Kaneesamkandi [119]; Mu’azu et al. [96]; Natour [125]; Nizami et al. [115]; Ouda et al. [112]; Rehan et al. [102]; Rehan et al. [111]; Seguela et al. [109]; Shahzad et al. [110]; Waqas et al. [103]; Waqas et al. [104]; Waqas et al. [105]

## References

[1] HLPE (2014). Food Losses and Waste in the Context of Sustainable Food Systems. A Report by the High Level Panel of Experts on Food Security and Nutrition of the Committee on World Food Security.

[2] FAO (2011). Global Food Losses and Food Waste: Extent, Causes and Prevention.

[3] FAO (2019). The State of Food and Agriculture 2019. Moving Forward on Food Loss and Waste Reduction.

[4] Lundqvist J., Martínez-Cortina L., Garrido G., López-Gunn L. (2010). Producing more or wasting less? Bracing the food security challenge of unpredictable rainfall. Re-Thinking Water and Food Security: Fourth Marcelino Botín Foundation Water Workshop.

[5] Parfitt J., Barthel M., Macnaughton S. (2010). Food waste within food supply chains: Quantification and potential for change to 2050. Philos. Trans. R. Soc. B Biol. Sci..

[6] Chalak A., Abou-Daher C., Chaaban J., Abiad M.G. (2016). The global economic and regulatory determinants of household food waste generation: A cross-country analysis. Waste Manag..

[7] WRAP (2008). The Food We Waste.

[8] WRAP (2012). Household Food and Drink Waste in the UK 2012.

[9] WRAP (2020). Food Waste Trends Survey 2019: Citizen Behaviours, Attitudes and Awareness around Food Waste.

[10] Stuart T. (2009). Waste: Uncovering the Global Food Scandal.

[11] FAO, IFAD, UNICEF, WFP, WHO (2019). The State of Food Security and Nutrition in the World 2019—Safeguarding Against Economic Slowdowns and Downturns.

[12] Serafini M., Toti E. (2016). Unsustainability of Obesity: Metabolic Food Waste. Front. Nutr..

[13] Smil V. (2004). Improving Efficiency and Reducing Waste in Our Food System. Environ. Sci..

[14] FAO (2017). The Future of Food and Agriculture: Trends and Challenges.

[15] Kummu M., de Moel H., Porkka M., Siebert S., Varis O., Ward P.J. (2012). Lost food, wasted resources: Global food supply chain losses and their impacts on freshwater, cropland, and fertiliser use. Sci. Total Environ..

[16] WRI (2013). Creating a Sustainable Food Future: A Menu of Solutions to Sustainably Feed more than 9 Billion People by 2050.

[17] Lipinski B., Hanson C., Lomax J., Kitinoja L., Waite R., Searchinger T., Craig H., Lomax J., Lisa K., Richard W. (2016). Reducing Food Loss and Waste.

[18] Spiker M.L., Hiza H.A.B., Siddiqi S.M., Neff R.A. (2017). Wasted Food, Wasted Nutrients: Nutrient Loss from Wasted Food in the United States and Comparison to Gaps in Dietary Intake. J. Acad. Nutr. Diet..

[19] UNEP (2012). The Critical Role of Global Food Consumption Patterns in Achieving Sustainable Food Systems and Food for all. A UNEP Discussion Paper.

[20] FAO (2013). Food Wastage Footprint. Impacts on Natural Resources.

[21] Bellù L.G. (2016). Food Losses and Waste: Issues and Policy Options.

[22] FAO. Key Facts on Food Loss and Waste You Should Know!.

[23] Nellemann C., MacDevette M., Manders T., Eickhout B., Svihus B., Prins A.G., Kaltenborn B.P. (2009). The Environmental Food Crisis—The Environment’s Role in Averting Future Food Crises. A UNEP Rapid Response Assessment. United Nations Environment Programme.

[24] Lee U., Han J., Wang M. (2017). Evaluation of landfill gas emissions from municipal solid waste landfills for the life-cycle analysis of waste-to-energy pathways. J. Clean. Prod..

[25] Zhang C., Xu T., Feng H., Chen S. (2019). Greenhouse Gas Emissions from Landfills: A Review and Bibliometric Analysis. Sustainability.

[26] Garnett T. (2011). Where are the best opportunities for reducing greenhouse gas emissions in the food system (including the food chain)?. Food Policy.

[27] Vanham D., Bidoglio G. (2013). A review on the indicator water footprint for the EU28. Ecol. Indic..

[28] Chapagain A., James K., Kosseva M., Webb C. (2013). Accounting for the impact of food waste on water resources and climate change. Food Industry Wastes: Assessment and Recuperation of Commodities.

[29] Wirsenius S., Azar C., Berndes G. (2010). How much land is needed for global food production under scenarios of dietary changes and livestock productivity increases in 2030?. Agric. Syst..

[30] Galli A., Iha K., Halle M., El Bilali H., Grunewald N., Eaton D., Capone R., Debs P., Bottalico F. (2017). Mediterranean countries’ food consumption and sourcing patterns: An Ecological Footprint viewpoint. Sci. Total Environ..

[31] Grizzetti B., Pretato U., Lassaletta L., Billen G., Garnier J. (2013). The contribution of food waste to global and European nitrogen pollution. Environ. Sci. Policy.

[32] FAO (2013). Food Wastage Footprint. Impacts on Natural Resources. Summary Report.

[33] Lundqvist J., Fraiture C.D., Molden D. (2008). Saving Water: From Field to Fork. Curbing Losses and Wastage in the Food Chain.

[34] Hall K.D., Guo J., Dore M., Chow C.C. (2009). The Progressive Increase of Food Waste in America and Its Environmental Impact.

[35] Lipinski B., Hanson C., Lomax J., Kitinoja L., Waite R., Searchinger T. (2016). Reducing Food Loss and Waste. Working Paper, Installment 2 of “Creating a Sustainable Food Future”.

[36] Rutten M.M. (2013). What Economic theory tells us about the impacts of reducing food losses and/or waste: Implications for research, policy and practice. Agric. Food Secur..

[37] European Commission Directive 2008/98/EC on Waste (Waste Framework Directive). http://ec.europa.eu/environment/waste/framework.

[38] Mourad M. (2016). Recycling, recovering and preventing “food waste”: Competing solutions for food systems sustainability in the United States and France. J. Clean. Prod..

[39] United States Environmental Protection Agency Food Recovery Hierarchy. https://www.epa.gov/sustainable-management-food/food-recovery-hierarchy.

[40] Capone R., El Bilali H., Debs D., Bottalico F., Cardone G., Berjan S., Elmenofi G.A.G., Aboubdillah A., Charbel L., Ali Arous S. (2016). Bread and Bakery Products Waste in Selected Mediterranean Arab Countries. Am. J. Food Nutr..

[41] Berjan S., Capone R., Debs P., El Bilali H. (2018). Food losses and waste: A global overview with a focus on Near East and North Africa region. Int. J. Agric. Manag. Dev..

[42] FAO (2015). Regional Overview of Food Insecurity—Near East and North Africa: Strengthening Regional Collaboration to Build Resilience for Food Security and Nutrition.

[43] FAO (2015). Regional Strategic Framework—Reducing Food Losses and Waste in the Near East & North Africa Region.

[44] Standing Committee for Economic and Commercial Cooperation of the Organization of Islamic Cooperation (2017). Reducing Food Waste in the OIC Countries.

[45] Abiad M.G., Meho L.I. (2018). Food loss and food waste research in the Arab world: A systematic review. Food Secur..

[46] Baig M.B., Gorski I., Neff R.A. (2019). Understanding and addressing waste of food in the Kingdom of Saudi Arabia. Saudi J. Biol. Sci..

[47] FAO (2014). Reducing Food Loss and Waste in the Near East and North Africa.

[48] INRA (2015). Addressing Agricultural Import Dependence in the Middle East—North Africa Region through to the Year 2050. Short Summary of the Final Report of a Study Carried Out by INRA (National Institute for Agronomic Research) for Pluriagri association.

[49] FAO, IFAD, WFP (2015). The State of Food Insecurity in the World 2015. Meeting the 2015 International Hunger Targets: Taking Stock of Uneven Progress.

[50] Ben Hassen T., El Bilali H. (2019). Food Security in the Gulf Cooperation Council Countries: Challenges and Prospects. J. Food Secur..

[51] The Economist Intelligence Unit (2020). Regional Report: Middle East and Africa. Global Food Security Index 2019.

[52] Samara A., Andersen P.T., Aro A.R. (2019). Health Promotion and Obesity in the Arab Gulf States: Challenges and Good Practices. J. Obes..

[53] Ali Arous S., Capone R., Debs P., Haddadi Y., El Bilali H., Bottalico F., Hamidouche M. (2017). Exploring household food waste issue in Algeria. AGROFOR Int. J..

[54] Elmenofi G., Capone R., Waked S., Debs P., Bottalico F., El Bilali H. An exploratory survey on household food waste in Egypt. Proceedings of the VI International Scientific Agriculture Symposium “Agrosym 2015”.

[55] Charbel L., Capone R., Grizi L., Debs P., Khalife D., El Bilali H., Bottalico F. (2016). Preliminary Insights on Household Food Wastage in Lebanon. J. Food Secur..

[56] Abouabdillah A., Capone R., El Youssfi L., Debs P., Harraq A., El Bilali H., El Amrani M., Bottalico F., Driouech N. (2015). Household food waste in Morocco: An exploratory survey. Proceedings of the Sixth International Scientific Agricultural Symposium “Agrosym 2015”.

[57] Sassi K., Capone R., Abid G., Debs P., El Bilali H., Daaloul Bouacha O., Bottalico F., Driouech N., Sfayhi Terras D. (2016). Food wastage by Tunisian households. Int. J. AgroFor.

[58] Moher D., Liberati A., Tetzlaff J., Altman D.G. (2009). Preferred Reporting Items for Systematic Reviews and Meta-Analyses: The PRISMA Statement. PLoS Med..

[59] El Bilali H. (2018). Transition heuristic frameworks in research on agro-food sustainability transitions. Environ. Dev. Sustain..

[60] El Bilali H. (2019). Research on agro-food sustainability transitions: Where are food security and nutrition?. Food Secur..

[61] Baig M.B., Al-Zahrani K.H., Schneider F., Straquadine G.S., Mourad M. (2019). Food waste posing a serious threat to sustainability in the Kingdom of Saudi Arabia—A systematic review. Saudi J. Biol. Sci..

[62] Anjum M., Miandad R., Waqas M., Ahmad I., Omar Z., Alafif A., Aburiazaiza A.S., Barakat M.A.E., Akhtar T. (2016). Solid waste management in Saudi Arabia: A review. J. Appl. Agric. Biotechnol..

[63] Qu X., Zhao Y., Yu R., Li Y., Falzone C., Smith G., Ikehata K. (2016). Health Effects Associated with Wastewater Treatment, Reuse, and Disposal. Water Environ. Res..

[64] Monchot H., Bailon S., Schiettecatte J. (2014). Archaeozoological evidence for traditional consumption of spiny-tailed lizard (Uromastyx aegyptia) in Saudi Arabia. J. Archaeol. Sci..

[65] Abdallah M., Shanableh A., Arab M., Shabib A., Adghim M., El-Sherbiny R. (2019). Waste to energy potential in middle income countries of MENA region based on multi-scenario analysis for Kafr El-Sheikh Governorate, Egypt. J. Environ. Manag..

[66] Chalak A., Abiad M.G., Diab M., Nasreddine L. (2019). The Determinants of Household Food Waste Generation and its Associated Caloric and Nutrient Losses: The Case of Lebanon. PLoS ONE.

[67] Al-Khatib I.A., Arafat H.A. (2010). A review of residential solid waste management in the occupied Palestinian Territory: A window for improvement?. Waste Manag. Res..

[68] Taghipour H., Mosaferi M. (2009). Characterization of medical waste from hospitals in Tabriz, Iran. Sci. Total Environ..

[69] Skourides I., Smith S.R., Loizides M. (2008). Sources and factors controlling the disposal of biodegradable municipal solid waste in urban and rural areas of Cyprus. Waste Manag. Res..

[70] Zaręba A., Krzemińska A., Łach J. (2017). Energy sustainable cities. From eco villages, eco districts towards zero carbon cities. E3S Web Conf..

[71] Abd-Rabboh H.S.M., Fawy K.F., Awwad N.S. (2019). Removal of copper(II) from aqueous samples using natural activated hydroxyapatite sorbent produced from camel bones. Desalin. Water Treat..

[72] Fitton N., Alexander P., Arnell N., Bajzelj B., Calvin K., Doelman J., Gerber J.S., Havlik P., Hasegawa T., Herrero M. (2019). The vulnerabilities of agricultural land and food production to future water scarcity. Glob. Environ. Chang..

[73] Sodiq A., Baloch A.A.B., Khan S.A., Sezer N., Mahmoud S., Jama M., Abdelaal A. (2019). Towards modern sustainable cities: Review of sustainability principles and trends. J. Clean. Prod..

[74] Pettett C.E., Al-Hajri A., Al-Jabiry H., Macdonald D.W., Yamaguchi N. (2018). A comparison of the Ranging behaviour and habitat use of the Ethiopian hedgehog (Paraechinus aethiopicus) in Qatar with hedgehog taxa from temperate environments. Sci. Rep..

[75] Alajmi F., Somerset S.M. (2015). Food system sustainability and vulnerability: Food acquisition during the military occupation of Kuwait. Public Health Nutr..

[76] Al-Doush I.I., Mahier T.J., Al Tufail M., Bogusz M.J. (2012). Occurrence of osthole in commonly available citrus fruits analyzed with GC-MS and LC-QTOF-MS. Int. J. Appl. Res. Nat. Prod..

[77] Carson G. Middle Eastern Promise Lures UK Packagers. https://www.packagingnews.co.uk/?s=Middle+Eastern+promise+lures+UK+packagers.

[78] Sengar A.S., Rawson A., Muthiah M., Kalakandan S.K. (2020). Comparison of different ultrasound assisted extraction techniques for pectin from tomato processing waste. Ultrason. Sonochem..

[79] Di Donato P., Taurisano V., Poli A., Gomez d’Ayala G., Nicolaus B., Malinconinco M., Santagata G. (2020). Vegetable wastes derived polysaccharides as natural eco-friendly plasticizers of sodium alginate. Carbohydr. Polym..

[80] Yahya N.A., Wahab R.A., Xine T.L.S., Hamid M.A. Ultrasound-assisted extraction of polyphenols from pineapple skin. Proceedings of the 2nd International Conference on Biosciences and Medical Engineering (ICBME2019)—AIP Conference Proceedings 2155.

[81] Pollini L., Rocchi R., Cossignani L., Mañes J., Compagnone D., Blasi F. (2019). Phenol Profiling and Nutraceutical Potential of Lycium spp. Leaf Extracts Obtained with Ultrasound and Microwave Assisted Techniques. Antioxidants.

[82] Nazeri M.A., Mat Zain N. (2018). Effect of Different Operating Parameters on Extraction of Active Compounds from Pitaya Peel by Microwave Assisted Extraction (MAE). J. Teknol..

[83] Grassino A.N., Brnčić M., Vikić-Topić D., Roca S., Dent M., Brnčić S.R. (2016). Ultrasound assisted extraction and characterization of pectin from tomato waste. Food Chem..

[84] Paini M., Casazza A.A., Aliakbarian B., Perego P., Binello A., Cravotto G. (2016). Influence of ethanol/water ratio in ultrasound and high-pressure/high-temperature phenolic compound extraction from agri-food waste. Int. J. Food Sci. Technol..

[85] Barba F.J., Puértolas E., Brnčić M., Panchev I.N., Dimitrov D.A., Athès-Dutour V., Moussa M., Souchon I. (2015). Emerging extraction. Food Waste Recovery.

[86] Marashlian N., El-Fadel M. (2005). The effect of food waste disposers on municipal waste and wastewater management. Waste Manag. Res..

[87] Adhikari B.K., Barrington S., Martinez J., King S. (2008). Characterization of food waste and bulking agents for composting. Waste Manag..

[88] Wheeler D.L. (2015). Food Security, Obesity, and the Politics of Resource Strain in Kuwait. World Med. Heal. Policy.

[89] Al-Yaqout A.F., Hamoda M.F. (2002). Report: Management problems of solid waste landfills in Kuwait. Waste Manag. Res..

[90] Al.Ansari M.S. (2012). Improving Solid Waste Management in Gulf Co-operation Council States: Developing Integrated Plans to Achieve Reduction in Greenhouse Gases. Mod. Appl. Sci..

[91] Al-Maaded M., Madi N.K., Kahraman R., Hodzic A., Ozerkan N.G. (2012). An Overview of Solid Waste Management and Plastic Recycling in Qatar. J. Polym. Environ..

[92] Elkhalifa S., Al-Ansari T., Mackey H.R., McKay G. (2019). Food waste to biochars through pyrolysis: A review. Resour. Conserv. Recycl..

[93] Demirbas A., Taylan O., Kaya D. (2016). Biogas production from municipal sewage sludge (MSS). Energy Sources Part A Recover. Util. Environ. Eff..

[94] Abdelaal A.H., McKay G., Mackey H.R. (2019). Food waste from a university campus in the Middle East: Drivers, composition, and resource recovery potential. Waste Manag..

[95] Elkhalifa S., AlNouss A., Al-Ansari T., Mackey H.R., Parthasarathy P., Mckay G. (2019). Simulation of Food Waste Pyrolysis for the Production of Biochar: A Qatar Case Study. Comput. Aided Chem. Eng..

[96] Mu’azu N.D., Blaisi N.I., Naji A.A., Abdel-Magid I.M., AlQahtany A. (2019). Food waste management current practices and sustainable future approaches: A Saudi Arabian perspectives. J. Mater. Cycles Waste Manag..

[97] Abdallah M., Shanableh A., Shabib A., Adghim M. (2018). Financial feasibility of waste to energy strategies in the United Arab Emirates. Waste Manag..

[98] Aktas E., Sahin H., Topaloglu Z., Oledinma A., Huda A.K.S., Irani Z., Sharif A.M., van’t Wout T., Kamrava M. (2018). A consumer behavioural approach to food waste. J. Enterp. Inf. Manag..

[99] Bennbaia S., Wazwaz A., Abujarbou A., Abdella G.M., Musharavati F. Towards sustainable society: Design of food waste recycling machine. Proceedings of the International Conference on Industrial Engineering and Operations Management.

[100] Bennbaia S., Wazwaz A., Abujarbou A. Towards sustainable society: Design of food waste recycling machine. Proceedings of the International Conference on Industrial Engineering and Operations Management.

[101] Irani Z., Sharif A.M., Lee H., Aktas E., Topaloğlu Z., van’t Wout T., Huda S. (2018). Managing food security through food waste and loss: Small data to big data. Comput. Oper. Res..

[102] Rehan M., Gardy J., Demirbas A., Rashid U., Budzianowski W.M., Pant D., Nizami A.S. (2018). Waste to biodiesel: A preliminary assessment for Saudi Arabia. Bioresour. Technol..

[103] Waqas M., Nizami A.S., Aburiazaiza A.S., Barakat M.A., Rashid M.I., Ismail I.M.I. (2018). Optimizing the process of food waste compost and valorizing its applications: A case study of Saudi Arabia. J. Clean. Prod..

[104] Waqas M., Almeelbi T., Nizami A.-S. (2018). Resource recovery of food waste through continuous thermophilic in-vessel composting. Environ. Sci. Pollut. Res..

[105] Waqas M., Nizami A.S., Aburiazaiza A.S., Barakat M.A., Ismail I.M.I., Rashid M.I. (2018). Optimization of food waste compost with the use of biochar. J. Environ. Manage..

[106] Alhajhoj M.R. (2017). Effects of different types of vermicompost on the growth and rooting characteristics of three rose rootstocks. J. Food Agric. Environ..

[107] Baawain M., Al-Mamun A., Omidvarborna H., Al-Amri W. (2017). Ultimate composition analysis of municipal solid waste in Muscat. J. Clean. Prod..

[108] Al-Ali Mustafa S.A.S.A.A. (2017). Growing food pyramids in the sand: How sustainable are Qatar’s self-sufficiency and foreign agro-investment policies?. J. Agric. Environ. Int. Dev..

[109] Seguela G., Littlewood J.R., Karani G. (2017). Onsite Food Waste Processing as an Opportunity to Conserve Water in a Medical Facility Case Study, Abu Dhabi. Energy Procedia.

[110] Shahzad K., Nizami A.S., Sagir M., Rehan M., Maier S., Khan M.Z., Ouda O.K.M., Ismail I.M.I., BaFail A.O. (2017). Biodiesel production potential from fat fraction of municipal waste in Makkah. PLoS ONE.

[111] Rehan M., Nizami A.-S., Asam Z.-Z., Ouda O.K.M., Gardy J., Raza G., Naqvi M., Mohammad Ismail I. (2017). Waste to Energy: A Case Study of Madinah City. Energy Procedia.

[112] Ouda O.K.M., Raza S.A., Nizami A.S., Rehan M., Al-Waked R., Korres N.E. (2016). Waste to energy potential: A case study of Saudi Arabia. Renew. Sustain. Energy Rev..

[113] Pirani S.I., Arafat H.A. (2016). Reduction of food waste generation in the hospitality industry. J. Clean. Prod..

[114] Pirani S.I., Arafat H.A. (2016). Interplay of food security, agriculture and tourism within GCC countries. Glob. Food Sec..

[115] Nizami A.-S., Rehan M., Ouda O.K.M., Shahzad K., Sadef Y., Iqbal T., Ismail I.M.I. (2015). An argument for developing waste-to-energy technologies in Saudi Arabia. Chem. Eng. Trans..

[116] Seed B. (2015). Sustainability in the Qatar national dietary guidelines, among the first to incorporate sustainability principles. Public Health Nutr..

[117] Al-Zahrani K., Baig M. (2014). Food Waste in the Kingdom of Saudi Arabia: Need for Extension Education Programs to Increase Public Awareness. Proceedings of the 10th International Academic Conferences.

[118] Amara A.A., Hamdan S., Melibary N. (2013). Management of food in the hajj in line with the rationalization of consumption and preservation of the environment. Majallat Ᾱlam al-Tarbiyah.

[119] Khan M.S.M., Kaneesamkandi Z. (2013). Biodegradable waste to biogas: Renewable energy option for the Kingdom of Saudi Arabia. Int. J. Innov. Appl. Stud..

[120] Aljamal A., Bagnied M. (2012). Food Consumption and Waste in Kuwait: The Prospects for Demand-Side Approach to Food Security. Int. Rev. Bus. Res. Pap..

[121] Alruqai I.M. (2012). Environmental Advantage Assessment of Recycling Food Waste in Riyadh, Saudi Arabia. Res. J. Environ. Sci..

[122] Abu Qdais H. (1997). Analysis of residential solid waste at generation sites. Waste Manag. Res..

[123] Al-Othman A.A., Hewedy F.M. (1997). Dietary Assessment of Male Students: A Study of what they wasted while in Residence. Nutr. Health.

[124] Al-Shoshan A.A. (1992). Study of the regular diet of selected hospitals of the Ministry of Health in Saudi Arabia: Edible plate waste and its monetary value. J. R. Soc. Health.

[125] Natour R.M. (1987). Utilization of municipal compost in Kuwait. Dirasat.

[126] Hsieh H.F., Shannon S.E. (2005). Three approaches to qualitative content analysis. Qual. Health Res..

[127] Petticrew M., Roberts H. (2008). Systematic Reviews in the Social Sciences.

[128] FAO (2013). Report of the Expert Consultation Meeting on Food Losses and Waste Reduction in the Near East Region: Towards a Regional Comprehensive Strategy.

[129] Alshawaf M. (2008). Evaluating the Economic and Environmental Impacts of Water Subsidies in Kuwait.

[130] Barilla Center for Food and Nutrition Food Sustainability Index. https://foodsustainability.eiu.com.

[131] Abdel Gelil I., Saab N., AFED (2015). Arab Environment: Sustainable Consumption. Annual Report of Arab Forum for Environment and Development 2015.

[132] Hwalla N., Bahn R.A., El Labban S., Abdel Gelil I., Saab N. (2015). Sustainable Food Consumption in Arab Countries. Arab Environment: Sustainable Consumption.

[133] Ericksen P.J. (2008). Conceptualizing food systems for global environmental change research. Glob. Environ. Chang..

[134] Ingram J.S.I. (2011). From Food Production to Food Security: Developing Interdisciplinary, Regional-Level Research.

[135] World Economic Forum (2014). Towards the Circular Economy: Accelerating the Scale-Up Across Global Supply Chains.

[136] Quested T.E., Marsh E., Stunell D., Parry A.D. (2013). Spaghetti soup: The complex world of food waste behaviours. Resour. Conserv. Recycl..

[137] Al-Turki A.I. (2010). Quality Assessment of Commercially Produced Composts in Saudi Arabia Market. Int. J. Agric. Res..

[138] Knoema Expenditures Spent on Food. https://knoema.com/ESFUSDA2010/expenditures-spent-on-food-by-selected-countries.

[139] UNDP, FAO (1973). Reconnaissance Soil Survey and Land Classification 1 Qatar.

[140] FAO, ITPS (2015). Status of the World’s Soil Resources (SWSR)—Main Report.

[141] Nordic Council of Ministers (2017). Preventing Food Waste—Better Use of Resources. Policy Brief.

[142] Saudi Grains Organization (SAGO) (2019). Saudi FLW Baseline—Food Loss & Waste Index in Kingdom of Saudi Arabia.

[143] Kingdom of Saudi Arabia (2019). Overview of Saudi Arabia’s 2020 G20 Presidency—Realizing Opportunities of the 21st Century for All.

